# Supplementary figures and images for: The iron–sulfur helicase DDX11 promotes the generation of single-stranded DNA for CHK1 activation
Source: Life Sci Alliance. 2020 Feb 18;3(3):e201900547. doi: 10.26508/lsa.201900547 (PMC7032568; doi:10.26508/lsa.201900547)

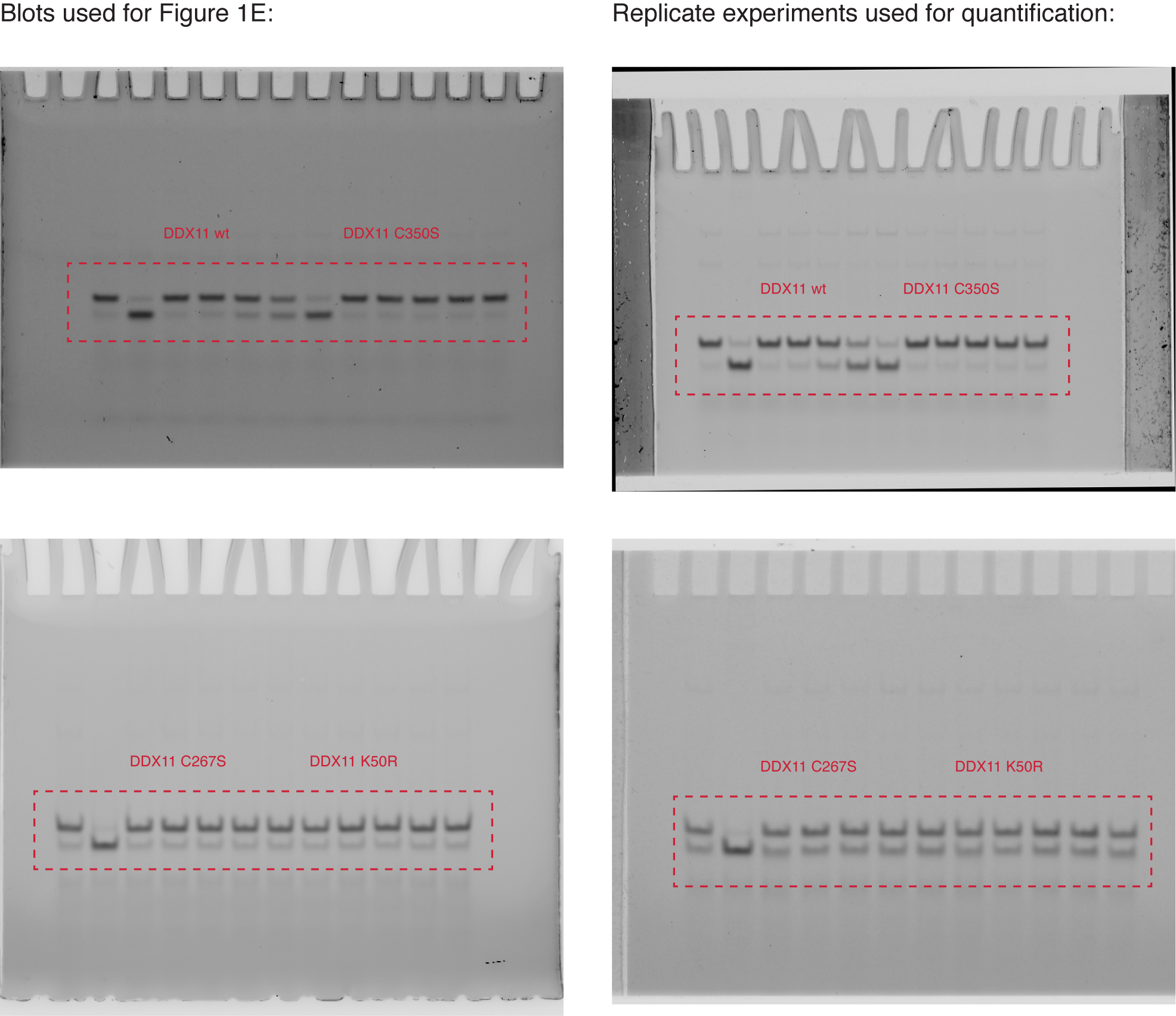

Supplement: Supplementary file 1 [file LSA-2019-00547_SdataF1.tif]
